# Short-Chain Fatty Acid Butyrate Induces Cilia Formation and Potentiates the Effects of HDAC6 Inhibitors in Cholangiocarcinoma Cells

**DOI:** 10.3389/fcell.2021.809382

**Published:** 2022-01-13

**Authors:** Kishor Pant, Seth Richard, Sergio A. Gradilone

**Affiliations:** ^1^ The Hormel Institute, University of Minnesota, Austin, MN, United States; ^2^ Masonic Cancer Center, University of Minnesota, Minneapolis, MN, United States

**Keywords:** short-chain fatty acid, butyrate, HDAC6, cilia, cholangiocarcinoma

## Abstract

Cholangiocarcinoma (CCA) is a deadly form of liver cancer with limited therapeutic approaches. The pathogenesis of CCA involves the loss of primary cilia in cholangiocytes, an important organelle that regulates several key cellular functions including the regulation of cell polarity, growth, and differentiation, by a mechanism involving increased expression of deacetylases like HDAC6 and SIRT1. Therefore, cilia restoration may represent an alternative and novel therapeutic approach against CCA. Butyrate is produced by bacterial fermentation of fibers in the intestine and has been shown to inhibit SIRT1, showing antitumor effects on various cancers. Herein, we investigated the role of butyrate on CCA cell proliferation, migration, and EMT and evaluated the synergistic effects with specific HDAC6 inhibition. When CCA cells, including HuCCT1 and KMCH, were treated with butyrate, the cilia formation and acetylated-tubulin levels were increased, while no significant effects were observed in normal human cholangiocytes. Butyrate treatment also depicted reduced cell proliferation in HuCCT1 and KMCH cells, but on the other hand, it affected cell growth of the normal cholangiocytes only at high concentrations. In HuCCT1 cells, spheroid formation and cell migration were also halted by butyrate treatment. Furthermore, we found that butyrate augmented the previously described effects of HDAC6 inhibitors on CCA cell proliferation and migration by reducing the expression of CD44, cyclin D1, PCNA, Zeb1, and Vimentin. In summary, butyrate targets cancer cell growth and migration and enhances the anti-cancer effects of HDAC6 inhibitors in CCA cells, suggesting that butyrate may have therapeutic effects in CCA and other ciliopathies.

## Introduction

Malignancy of the bile ducts, also called cholangiocarcinoma (CCA), is a deadly form of liver cancer. CCA is the second most common primary liver malignancy in adults, accounting for 15–20% of cases. It is a rare form of cancer in the United States but occurs at high rates in Asian countries mainly in Thailand, China, and India. CCA is one of the deadly forms of cancer that progresses without symptoms until advanced stages and leaves patients with inadequate therapeutic choices (Banales et al., 2020). The pathogenesis of CCA, as well as in other tumors, seems to involve the loss of primary cilia in cholangiocytes (Gradilone et al., 2013; Razumilava et al., 2014; Gradilone et al., 2017; Merino-Azpitarte et al., 2017; Mansini et al., 2018, 2019; Banales et al., 2020; Pant et al., 2020; Peixoto et al., 2020a, 2020b). The primary cilium is an important organelle, present in almost every cell of the human body, that regulates a number of vital functions including sensing growth signals, liquid flow, osmotic pressure, and hormones (Gradilone et al., 2017; Mansini et al., 2018; Peixoto et al., 2020b). Importantly, primary cilia have been also reported to regulate the cell cycle and polarity in the cells of unicellular and multicellular organisms (Marshall, 2010). The mechanisms underlying the loss of cilia in CCA include the increased activity of deacetylases like HDAC6 and SIRT1, which removes the acetyl group from the α-tubulin in the ciliary axoneme in cholangiocytes and leads to cell proliferation, migration, and invasion in CCA (Gradilone et al., 2013; Pant et al., 2020, 2021). Therefore, cilia restoration could be advantageous as an effective therapeutic approach against CCA.

Butyrate is a four-carbon member of the short-chain fatty acids, produced during the fermentation of dietary fibers and complex carbohydrates by gut bacteria in the intestine (Louis et al., 2014). Butyrate is not only a source of energy but also identified as an HDAC inhibitor (Louis et al., 2014; Gomes et al., 2020). For instance, butyrate mainly can inhibit NAD-dependent Class III HDACs (SIRT family) and have some effects in Class IIa HDACs like HDAC4, 5, and 7 but do not affect Class IIb members like HDAC6 and HDAC10 (Corfe, 2012; Schilderink et al., 2013). Butyrate has been reported to inhibit tumor cell growth in colon cancer, HCC, pancreatic, and lung cancer cells (Farrow et al., 2003; Chopin et al., 2004; Donohoe et al., 2014, 2014; Pant et al., 2017a, 2017b). In addition, butyrate has been reported as an anti-inflammatory and antiviral agent (Pant et al., 2019; Gomes et al., 2020; Salvi and Cowles, 2021). Restoration of cilia using HDAC inhibitors, including ACY1215, Tubastatin-A, and sirtinol has been utilized to inhibit HDAC6 and SIRT1, respectively (Gradilone et al., 2013; Peixoto et al., 2020a; Pant et al., 2021). We have previously shown that inhibition of SIRT1 and HDAC6 induces cilia formation and inhibits cell growth in CCA cells (Gradilone et al., 2013; Pant et al., 2021). In this study, we assessed the role of butyrate in cilia formation in CCA cells and the effects on cell growth, migration, and EMT.

## Materials and Methods

### Cell Culture and Treatments

The CCA cell lines HuCCT1 and KMCH were cultured in Dulbecco’s Modified Eagle Medium (DMEM) (Life Technologies, Carlsbad, CA, USA) with 10% FBS and 1% penicillin/streptomycin. In addition, normal human cholangiocytes (H69 and NHC) were also used for this study. Different concentrations of butyrate (0–5 mM) and ACY1215 (0.5–2 μM) were assessed in the cells for 1–3 days and compared to corresponding vehicle controls (DMSO or water).

### shRNA Transfection

IFT88 and HDAC6 shRNA were stably transfected in cells with lentiviral-mediated vectors. Non-target (NT) shRNAs were used as controls. Cells were selected by adding puromycin (2 μg/ml) to the culture media.

### Western Blot Analysis

Cellular proteins were isolated by collecting cells with RIPA buffer*,* along with a protease inhibitor cocktail. An equal amount of protein (∼20 µg/well) was loaded in the gels, separated in 10% SDS-PAGE, and transferred to nitrocellulose membranes. BSA (5%) was used for blocking the membranes and then incubated with primary antibodies overnight at 4°C. Membranes were washed with Tris-buffered saline +0.1% Tween 20 (TBS-T) and incubated further with respective secondary antibodies. After washing, ECL Western blotting substrate (Thermo) was used to detect chemiluminescence and images developed using in AI600 imager.

### Immunofluorescence

HuCCT1 and H69 cells were grown in glass slides for 48 h and treated with butyrate (2 mM) or vehicle for 3 days in serum-free media. Primary antibodies against Ac-α-tubulin were used after fixation and blocking. Fluorescent detection was done through incubation with goat anti-mouse secondary antibody labeled with Alexa Fluor dye (Invitrogen/Life Technologies) for 1 h. Nuclei were marked with DAPI. The cilia were examined with a Zeiss Apotome.

### Cilia Frequency and Length Analysis

Primary cilia percentages were determined by physically calculating the number of cilia and total nuclei per slide. Ciliary length was calculated in at least five different fields by ImageJ software.

### IncuCyte Cell Growth Analysis

Around 1,000 cells per well were incubated into 96-well plates for 12 h before butyrate and/or ACY1215 treatments. Cells were incubated for at least 72 h in IncuCyte for cell growth analysis.

### MTS Assay

MTS assay was performed after the treatments of butyrate and ACY1215 for 48 h to measure the cell proliferation as described by manufacturer (Promega, Madison, WI, United States).

### 3D Spheroids Formation Assay

Approximately 2,000 cells were plated in 96-well round-bottom plates (Corning) and centrifuged at 1,500 rpm for 3 min. Plates were incubated in the IncuCyte live-cell analysis system and schedule for 4-h repeat scanning with 10x objective. Finally, the spheroid area was calculated after each image and indicated as bar graphs.

### Scratch Wound Assay

Scratch wound assays were performed in 96-well Essen ImageLock™ plates (Essen BioSciences, Ann Arbor, MI, USA); 2 × 10^5^ cells were seeded in each well and allowed to grow at 37°C until a confluent monolayer was formed. A scratch was made using the WoundMaker™ (Essen BioSciences) followed by drug treatment in fresh media. Images of these plates were captured with 4 h intervals, and relative wound area was measured with IncuCyte Live cell system.

### Statistical Analysis

All experiments were performed at least three times (*n* = 3). The data were expressed as mean ± standard error. Unpaired Student’s *t*-test between two groups and one-way ANOVA was performed among the groups. The data was considered statistically significant if p < 0.05.

## Results

### Butyrate Induces Cilia Formation in CCA Cells

**FIGURE 1:**
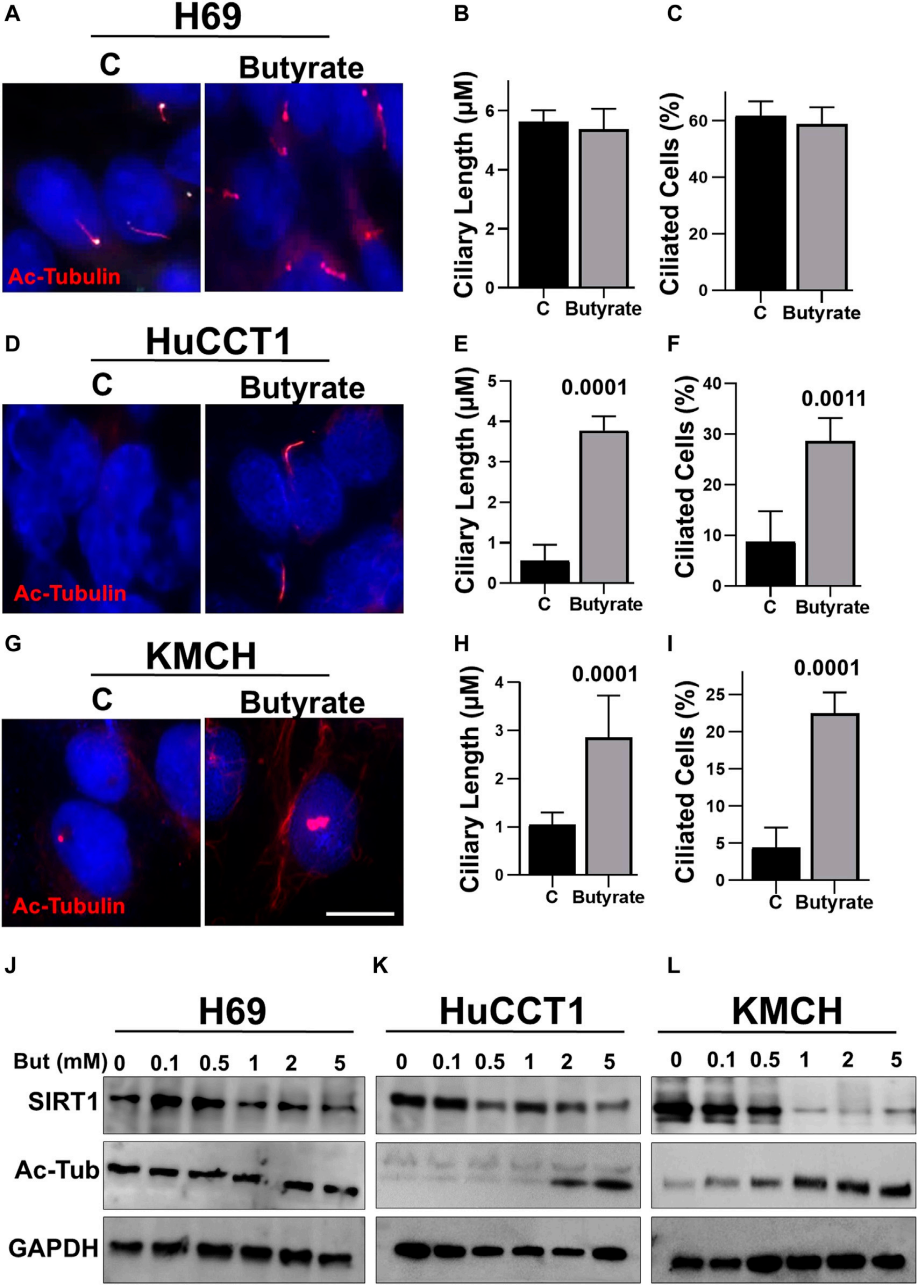
Butyrate induces cilia formation of CCA cells. Both HuCCT1 and H69 cells were cultured for 24 h and treated with butyrate (2 mM) or vehicle for 48 h in serum-free media. The cells were fixed and stained for the Ac-alpha-tubulin antibody. **(A)** The H69 cells were observed under microscope 63x oil for cilia images, and the representative picture is shown. **(B, C)** The cilia length and frequency were calculated in H69 cells. **(D)** HuCCT1 cells were visualized under microscope for cilia imaging. **(E, F)** The cilia length and frequency were measured in HuCCT1 cells. **(G)** KMCH cells were visualized under microscope for cilia imaging with Ac-tubulin staining. **(H, I)** The cilia length and frequency were measured in KMCH cells. **(J)** The expression of SIRT1 and Ac-tubulin was checked in H69 cell treated with butyrate (0.1–5 mM) using Western blotting. **(K, L)** Western blotting was done for the SIRT1 and Ac-tubulin in the presence of Butyrate in different concentrations (0.1–5 mM) in HuCCT1 and KMCH cells. GAPDH was used was loading control.

To assess the effects of butyrate treatments in cholangiocytes, we analyzed cilia formation in normal ciliated cell (H69) and CCA cells (HuCCT1 and KMCH). Butyrate treatment showed no effects on the cilia length and frequency in H69 cell (Figures 1A–C). However, HuCCT1 (Figures 1D–F) and KMCH cells (Figures 1G–I) showed significantly increased ciliary length and frequency upon butyrate treatment. Furthermore, we also found that butyrate treatment does not significantly affect SIRT1 protein expression and levels of Ac-α-tubulin in H69 cells (Figure 1J), but decreased SIRT1 expression and increased Ac-α-tubulin levels in HuCCT1 and KMCH cells in a concentration-dependent manner (Figures 1K,L). Taken together, these data suggest that butyrate inhibits SIRT1 expression, induces the acetylation of α-tubulin protein and induces cilia formation in CCA cells.

### Effects of Butyrate on CCA Cell Proliferation and Migration

**FIGURE 2:**
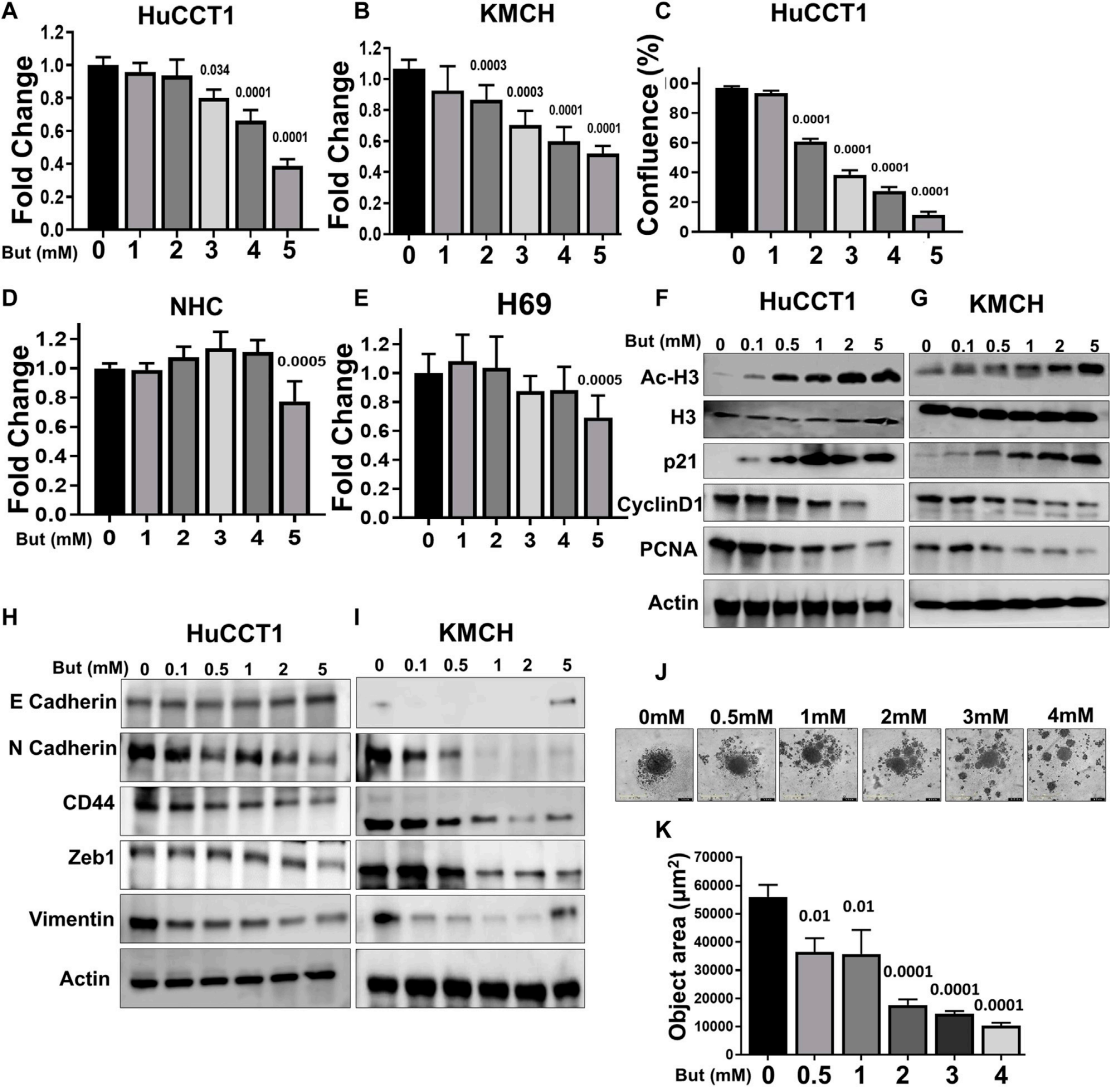
Effects of butyrate on CCA cell proliferation and migration. **(A, B)** MTS cell proliferation assay was done in HuCCT1 and KMCH cells after the treatment of butyrate (1–5 mM). **(C)** HuCCT1 cell proliferation was determined using the IncuCyte in the presence of butyrate (1–5 mM). **(D, E)** MTS cell proliferation assay was done in normal cholangiocytes (NHC and H69) after the treatment of butyrate (1–5 mM). **(F, G)** HuCCT1 and KMCH cells were treated with butyrate (0.1–5 mM) for 48 h, and Western blotting for Ac-histone H3, total histone, p21, cyclin D1, and PCNA were performed. β-Actin was used as a loading control. **(H, I)** The expression of E-Cadherin, N-Cadherin, CD44, Zeb1, and Vimentin using Western blots in butyrate-treated HuCCT1 and KMCH cells; β-actin was used as an internal loading control. **(J, K)** Spheroid formation assay was performed in the HuCCT1 cells after butyrate treatment, and spheroid area was determined using IncuCyte.

Next, we assessed the effects of butyrate on CCA cell proliferation using the IncuCyte live cell imaging and MTS assays and found decreased cell growth in both cells with increasing concentrations of butyrate (Figures 2A–C). Conversely, the butyrate treatment in two normal cholangiocyte cell lines (H69 and NHC) showed significant effect in cell growth only at the highest concentration (Figures 2D,E). Then, we assessed the effects of butyrate on some of the intracellular markers of cell growth (PCNA), cell cycle (Cyclin D1, p21), EMT control (E and N Cadherin, CD44, Zeb1, Vimentin), and epigenetic (Ac-H3) markers, and found that acetylation of histone H3 and the cell cycle inhibitor protein cyclin dependent kinase (CDK) inhibitor p21 expression were increased in both HuCCT1 and KMCH cells in a dose-dependent manner; on the contrary, expression of the cell cycle regulator Cyclin D1 (Karimian et al., 2016) and the proliferating cell nuclear antigen (PCNA) protein levels decreased with butyrate treatment (Figures 2F,G). Furthermore, the expression of the epithelial marker E-Cadherin increased but the expression of the mesenchymal markers N-Cadherin, CD44, Zeb1, and Vimentin (Marconi et al., 2021) were reduced with butyrate treatment in both CCA cell lines (Figures 2H,I). Finally, we analyzed the spheroid formation using IncuCyte and found that the area of spheroid was decreased with increasing concentration of butyrate in HuCCT1 cells (Figures 2J,K). Taken together, these data suggest that butyrate decreases CCA cell proliferation likely by mechanisms including epigenetics, cell cycle, and EMT regulation.

### Butyrate Potentiates the Effect of HDAC6 Inhibition

**FIGURE 3:**
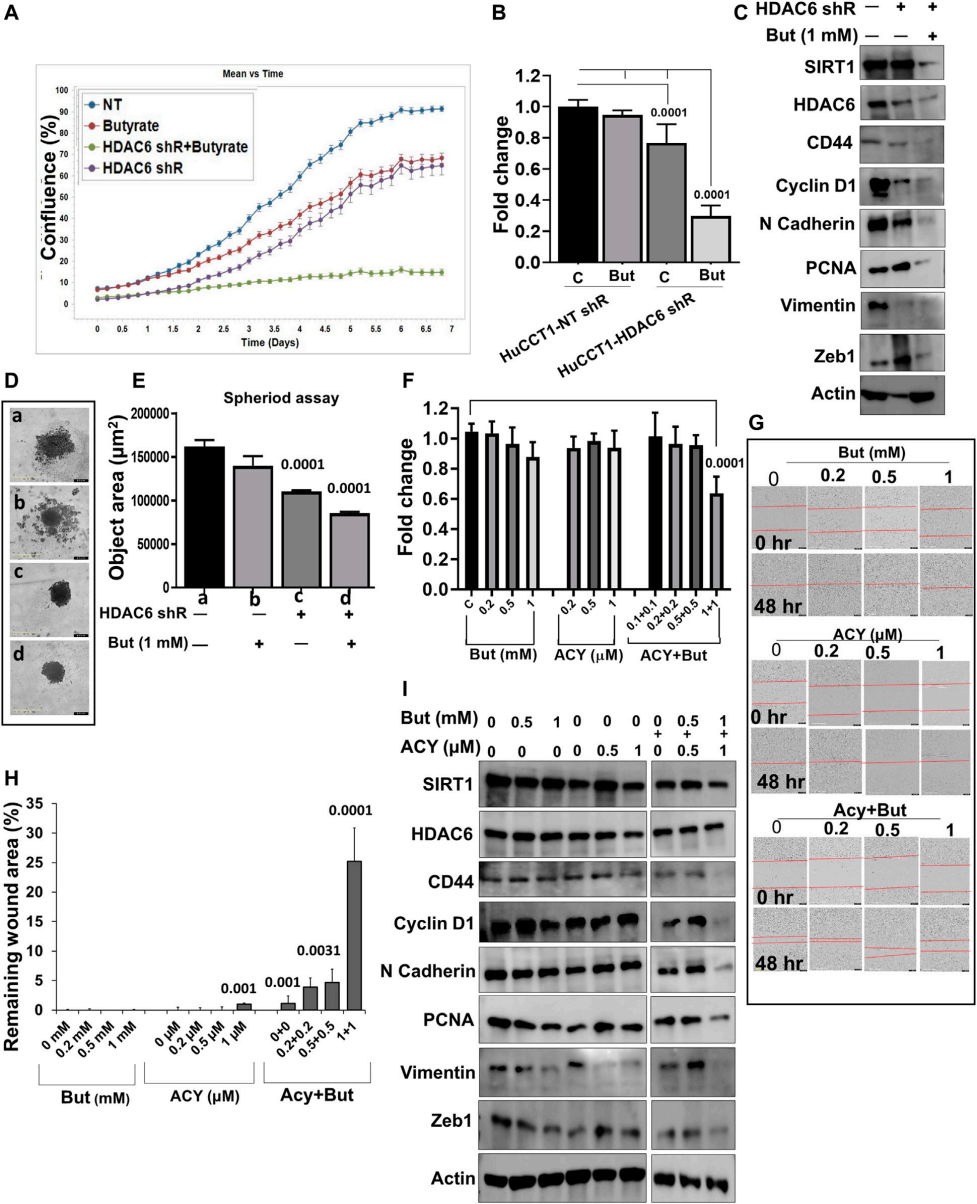
Butyrate potentiates the effect of the HDAC6 inhibitors. **(A)** IncyCyte cell proliferation assay was done in HuCCT1 cells transfected with HDAC6 shRNA and butyrate (1 mM), and non-target shRNA was used as a control. **(B)** MTS cell growth assay was done in HuCCT1-HDAC6 shRNA alone or in the presence of butyrate (1 mM). **(C)** Total cellular protein was isolated from HuCCT1 (NT, HDAC6-shR and HDAC6-shR + butyrate) cells and Western blots were performed for SIRT1, HDAC6, CD44, Cyclin D1, N-Cadherin, PCNA, Vimentin, Zeb1, and β-actin. **(D, E)** Spheroid assay was done using IncyCyte, and spheroid area was measured. **(F)** MTS assay was done in HuCCT1 cells in the presence of ACY1215 (0.5 and 1 μM) and butyrate (0.5 and 1 mM). **(G, H)** Scratch assay was analyzed using IncuCyte; HuCCT1 cells were treated with ACY1215 (0.2–1 μM) and butyrate (0.2 and 1 mM) for 48 h post-wound, and the remaining wound area was calculated. **(I)** Western blotting in HuCCT1 cells in the presence of ACY1215 (0.5 and 1 μM) and butyrate (0.5 and 1 mM) was done for SIRT1, HDAC6, CD44, Cyclin D1, N-Cadherin, PCNA, Vimentin, and Zeb1. β-actin was used as loading control.

As we previously showed that HDAC6 inhibition inhibits cell proliferation in a ciliary restoration-dependent manner (Gradilone et al., 2013) and that SIRT1 is also involved in this process (Pant et al., 2021), we now sought to assess the effect of the combined effects of butyrate with HDAC6 inhibition on HuCCT1 cells. First, we knocked down HDAC6 by specific shRNA stable transfections in HuCCT1 cells. Treatment with butyrate (1 mM) alone had minimal effects on HuCCT1-NT-shRNA cell proliferation but induced a significant growth arrest in the HuCCT1-HDAC6-shRNA cells (Figures 3A,B). We also noticed that butyrate (1 mM) treatment prompted the inhibition of SIRT1, HDAC6, CD44, Cyclin D1, N Cadherin, PCNA, Vimentin, and Zeb1 expression in HDAC6-shRNA knocked down cells (Figure 3C). Moreover, using the 3D spheroid assay, we found that butyrate treatment significantly decreased the spheroid size in HDAC6-shRNA cells, while minimally affecting the control NT-shRNA HuCCT1 cells (Figures 3D,E). In addition to the shRNA model, we also tested an HDAC6 specific inhibitor drug (ACY1215) alongside butyrate. We found that treatment with butyrate (0.2–1 mM) or ACY1215 (0.2–1 μM) alone did not affect cell proliferation, but the combinatorial treatment of butyrate with ACY1215 significantly inhibited the cell growth and migration (Figures 3F–H). Consistently, the expression of CD44, Cyclin D1, N-Cadherin, PCNA, Vimentin, and Zeb1 were decreased with the combined treatment of butyrate (0.5–1 mM) and ACY1215 (0.5–1 μM) (Figure 3I). All together, these results indicate that butyrate effectively hinders CCA cell growth in presence of HDAC6 inhibitors.

## Discussion

Elevated expression of HDAC family members like HDAC6 and SIRT1 has been associated with the loss of primary cilia and malignancy in CCA cells. Furthermore, the partial restoration of ciliary expression in CCA cells by inhibition of either HDAC6 or SIRT1 has been linked to decreased cell growth and migration in the tumor cells (Gradilone et al., 2013; Peixoto et al., 2020a, 2020b; Pant et al., 2021). We analyzed the relevance of the natural occurring short-chain fatty acid butyrate-induced cilia formation and the downstream effects in cell growth and the combinatorial effects with specific HDAC6 inhibition in CCA cells. The present study main findings include that butyrate treatment promotes cilia formation in CCA cells and inhibits cell growth likely by multifactorial mechanisms including epigenetics and EMT regulation. Furthermore, the use of combined butyrate and HDAC6 inhibition suggests a synergistic effect on cell proliferation, migration, and EMT.

Previously, butyrate has been reported as an anti-cancer agent in colon, pancreas, and hepatocellular cancers (Farrow et al., 2003; Donohoe et al., 2014; Pant et al., 2017a, 2017b). However, the potential effects of butyrate in CCA were unknown. Consistent with other tumor types previously described, we found that butyrate inhibits CCA cell growth at lower concentrations compared to normal cholangiocytes. Again, these observations are in agreement with previous results showing that butyrate prevents growth in colorectal cancer cells compared to normal cells (Donner, 2012; Donohoe et al., 2014; Wang et al., 2020). Furthermore, butyrate inhibits cell migration and invasion in HCC, bladder cancer, prostate cancer, and colorectal cancer cells (Zeng and Briske-Anderson, 2005; Mu et al., 2013, 1; Xu et al., 2018; Wang et al., 2020). In addition, butyrate treatment inhibited cancer cell migration via downregulation of HDACs including SIRT1 expression (Li et al., 2017; Cao et al., 2019). Therefore, it is possible that butyrate treatment regulates CCA cell migration and proliferation-associated proteins by lowering SIRT1 expression as our results showed.

Our data showed that butyrate can rescue ciliary expression in CCA cells, and consistent with our previous studies, the restoration of cilia is positively linked with CCA cell growth inhibition (Gradilone et al., 2013; Pant et al., 2021). However, it was previously described that butyrate also affects several signaling including, Akt/mTOR, MAP kinase, and Wnt pathways (Zhang et al., 2010; Lazarova et al., 2014; Pant et al., 2017b; Li et al., 2017; Cao et al., 2019). Consequently, the effects on CCA cell proliferation are likely due to a diverse range of targets beyond ciliary restoration as shown by the effects we observed on histone acetylation or even metabolic alteration as described in colorectal cancer cells (Donohoe et al., 2012; Li et al., 2018; Eslami et al., 2020). Interestingly, the use of butyrate in combination with HDAC6 inhibition showed increased effectiveness. The main reason for this synergistic effect is likely that butyrate can target both mitochondrial metabolism and HDACs in general (Donohoe et al., 2014; Wang et al., 2020).

Multiple lines of evidence have demonstrated that short-chain fatty acids, including butyrate, enhance the anticancer properties of other agents (Rosato et al., 2004, 2004; Singh and Lai, 2005; Yoon et al., 2011; Groh et al., 2015; Kobayashi et al., 2018). This study supports the anticancer effect of butyrate in combination with HDAC6 inhibitors in CCA cells in comparison to either agent alone. Since butyrate production in the gut can be induced by microbiota and/or dietary interventions, such manipulations may be a potential therapeutic or preventive approach for patients with CCA or at high risk for developing this type of cancer and warrant further investigations.

## Conclusion

**FIGURE 4:**
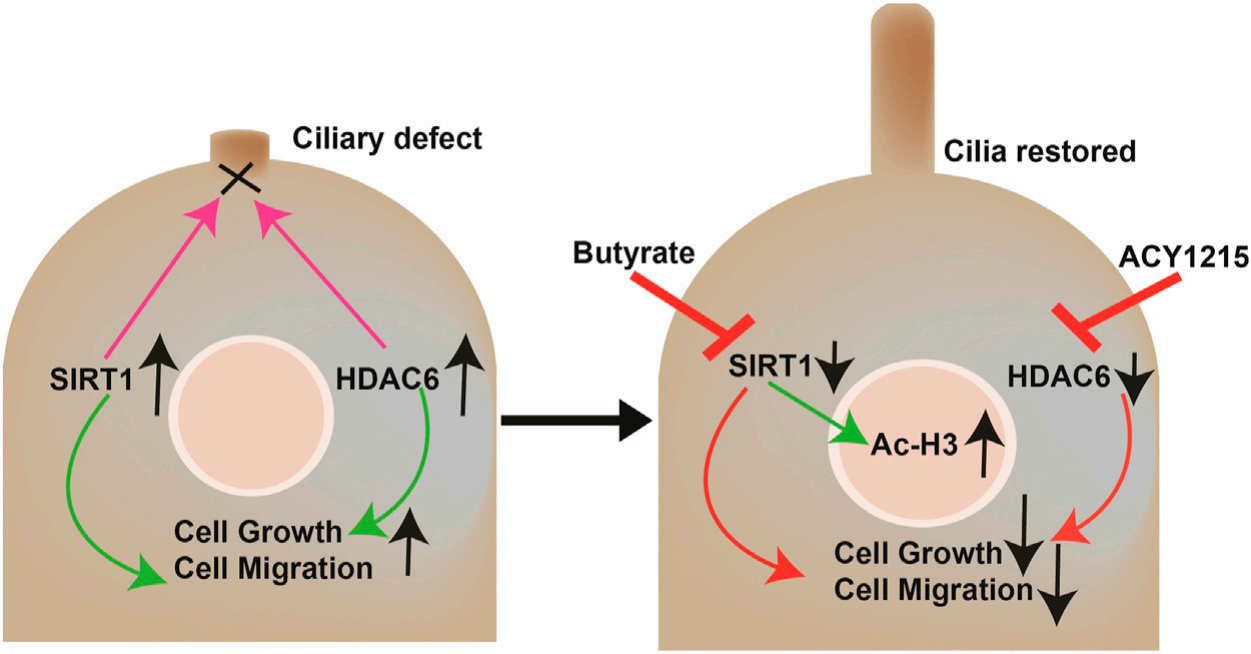
Working model. CCA cells show reduced ciliary expression due to the overexpression of the deacetylases HDAC6 and SIRT1 that target the ciliary axoneme among other functions. The loss of primary cilia induces cell proliferation, migration, and EMT. The combined treatment with butyrate and ACY-1215 synergistically decreases proliferation, migration, and EMT by directly restoring ciliary expression and affecting the signaling pathways downstream these organelles that normally repress cell growth, and indirectly by SIRT1-induced epigenetics regulation.

In summary, as described in Figure 4, our study showed that butyrate induces cilia formation and growth inhibition in CCA cells. Moreover, the combination of butyrate with HDAC6 inhibitors effectively halts cell growth, migration, and EMT in CCA cells at lower doses that may have no significant effects on normal healthy cells.

## Data Availability

The raw data supporting the conclusion of this article will be made available by the authors, without undue reservation.

## Abbreviations

BSA, bovine serum albumin; CCA, cholangiocarcinoma; cDNA, complementary DNA; EMT, epithelial–mesenchymal transition; gRNA, guide RNA; HCC, hepatocellular carcinoma; HDAC, histone deacetylase; NT, non-target; shRNA, short RNA.
